# Plastidial α-glucan phosphorylase 1 complexes with disproportionating enzyme 1 in *Ipomoea batatas* storage roots for elevating malto-oligosaccharide metabolism

**DOI:** 10.1371/journal.pone.0177115

**Published:** 2017-05-04

**Authors:** Yi-Chen Lin, Shih-Chung Chang, Rong-Huay Juang

**Affiliations:** Department of Biochemical Science and Technology, College of Life Science, National Taiwan University, Taipei, Taiwan; Shanghai Institutes for Biological Sciences, CHINA

## Abstract

It has been proposed that malto-oligosaccharides (MOSs) are possibly recycled back into amylopectin biosynthesis via the sequential reactions catalyzed by plastidial α-glucan phosphorylase 1 (Pho1) and disproportionating enzyme 1 (Dpe1). In the present study, the reciprocal co-immunoprecipitation experiments using specific antibodies against Pho1 and Dpe1 demonstrated that these two enzymes can form a complex (the PD complex) in *Ipomoea batatas* storage roots. The immunohistochemistry analyses also revealed the co-localization of Pho1 and Dpe1 in the amyloplasts, and the protein levels of Pho1 and Dpe1 increased gradually throughout sweet potato storage root development. A high molecular weight PD complex was co-purified from sweet potato storage root lysates by size exclusion chromatography. Enzyme kinetic analyses showed that the PD complex can catalyze maltotriose and maltotetraose to generate glucose-1-phosphate in the presence of inorganic phosphate, and it also performs greater Dpe1 activity toward MOSs than does free form Dpe1. These data suggest that Pho1 and Dpe1 may form a metabolon complex, which provides elevated metabolic fluxes for MOS metabolism via a direct transfer of sugar intermediates, resulting in recycling of glucosyl units back into amylopectin biosynthesis more efficiently.

## Introduction

Most agricultural plants store the carbon source derived from photosynthesis as starch in plastids. Starch consists of approximately 25% amylose and 75% amylopectin, and accumulates as a polymeric complex with a hierarchical order. Amylose is composed of unbranched α-1,4 linked glucans. Amylopectin contains a backbone of α-1,4 linked glucans and ~5% of α-1,6 branched glucans. The large, insoluble and semicrystalline granules of amylopectin, which allow efficient packing of large amounts of glucose (Glc), require the specific length of linear chains and the nonrandom location of branch linkages [1, 2].

Current understanding of starch biosynthesis involves at least four enzyme reactions. ADP-Glc pyrophosphorylase (AGPase) catalyzes the conversion of Glc-1-P to ADP-Glc. Starch synthases (SSs) use ADP-Glc as substrates to extend the α-1,4 linked glucans. Starch branching enzymes (BEs) are responsible for the formation of α-1,6 linked glucans. Starch debranching enzymes (DBEs), which hydrolyze α-1,6 linked glucans, are also involved in amylopectin biosynthesis [3]. In addition, genetic studies also implicate that plastidial α-glucan phosphorylase (Pho) [4, 5] and disproportionating enzyme (Dpe) [6–9] are potential enzymes involved in starch biosynthesis in plant storage tissues.

Two types of Pho (Pho1 and Pho2) are generally observed in higher plants. Pho1 and Pho2 differ in structure, enzyme kinetic property, subcellular localization, and their expression pattern during plant cell development [10]. The plastidial isoform of Pho (as known as Pho1, or L-SP, EC 2.4.1.1) possesses a high affinity toward linear malto-oligosaccharides (MOSs), whereas the cytosolic isoform of Pho (as known as Pho2 or H-SP) has a high affinity toward highly branched glucans. Both of Pho1 and Pho2 catalyze the reversible phosphorolysis of glucans and generate glucose-1-phosphate (Glc-1-P) as one of its products in the presence of inorganic phosphate. Since this reaction is reversible, the exact catalytic direction of Pho under physiological conditions is still controversial. Previous studies found that when MOSs composed of 4–7 glucose residues (G_4_ to G_7_) were used as substrates, Pho1 favored the phosphorolytic reaction over the synthetic reaction *in vitro* [11]. Dysfunction of the *Pho1* genes of *Chlamydomonas reinhardtii* and *Oryza sativa* significantly reduced the starch content and led to a modified amylopectin structure with the restricted degree of polymerization to less than 25 [4, 5]. Furthermore, it has been reported that Pho1 in the wild type of potato tubers (*Solanum tuberosum* L.) was involved in the formation of the short glucan chains when the potato plants were cultivated at low temperatures [12]. Our group also found that Pho1 is proteolytically modified by protein-protein interaction with the 20S proteasome under heat stress conditions [13]. These findings suggest that Pho1 might play versatile roles in starch metabolism under normal and stress conditions.

It has been shown that Pho1 forms multiprotein complexes with starch biosynthetic enzymes in different species. Pho1 interacts with starch branching enzymes (SBEI and SBEIIb) in wheat [14] and with starch synthases (GBSSI, SSI and SSIIa) and starch branching enzymes (SBEI and SBEIIa) in the amylose extender mutant of maize [15]. In starch branching enzyme (SBE) down-regulated lines (ΔSBEIIb) of barley, Pho1 is incorporated into a protein complex with SBEI and SBEIIa [16]. Pho1 also interacts with starch branching enzyme (SBEIIa) in rice endosperm [17]. These findings suggest that Pho1 might play certain roles in MOS metabolism and starch biosynthesis. To characterize the physiological function of Pho1 in the starch biosynthesis pathway in sweet potato storage roots, the co-immunoprecipitation and mass spectrometry analysis were applied to identify the potential Pho 1 interacting proteins. Here we found that disproportionating enzyme 1 (Dpe1, or D-enzyme, EC 2.4.1.25) is a Pho1 interacting protein.

Dpe1 is a plastidial α-1,4 glucanotransferase. It catalyzes the cleavage of α-1,4 glucans which are larger than maltotriose, and transfers glucosyl units to another glucan and releases glucose. Another disproportionating enzyme isoform in higher plants is a cytosolic α-1,4 glucanotransferase (named Dpe2), which metabolizes maltose by transferring a Glc moiety to a glycan polymer and releasing Glc [18]. A knockout mutant of *Dpe1* displayed a reduced starch degradation rate and largely accumulated MOSs, especially maltotriose, in *Arabidopsis* leave [19]. Repression of *Dpe1* expression in potato tubers led to higher MOS content [20] and showed no influence on starch degradation [21]. A mutation in the *Dpe1* gene of *C*. *reinhardtii* altered amylopectin’s structure to form polymeric chains with 3 to 16 Glc residues, and led to a substantial increase in MOS content and a reduction of granular starch deposition [6–8]. Overexpression of Dpe1 in rice endosperm affected the structure of starch granules and the accumulation of soluble MOSs [9]. These studies suggest that MOSs might be the primary substrates of Dpe1 *in vivo* and imply that Dpe1 may play a critical role in MOS metabolism.

Previous studies speculated that the actions of Dpe1 and Pho1 in starch metabolism are coordinated [6, 7, 22, 23]. In the glucan-trimming model [2], the branched glucans are removed from a precursor called pre-amylopectin by starch debranching enzymes, and the unordered structure of pre-amylopectin is subsequently converted to an ordered structure of amylopectin [1–3]. In this model, MOSs are proposed to be released by debranching enzymes, and Dpe1 could convert the short-chain MOSs to long-chain MOSs, which are suitable for phosphorolysis by Pho1 to generate Glc-1-P and then be converted to ADP-Glc for starch biosynthesis [2]. However, the direct evidence that Pho1 can function in conjunction with Dpe1 in recycling MOSs is still unclear.

Here we present evidence that Dpe1 associates with Pho1 in the amyloplast of *Ipomoea batatas* storage roots. The protein levels of the Pho1-Dpe1 complex (PD complex) increase during sweet potato storage root development, indicating that this complex may contribute to amylopectin biosynthesis. The enzyme kinetic analyses also revealed that the catalytic activity of the PD complex toward maltotetraose is higher than that of free form Dpe1. It has been suggested that sequential enzymes of a metabolic pathways might form complexes (termed metabolon) to directly transfer metabolic intermediates from one active site to another (termed metabolite channeling) [24]. In agreement with this concept, we speculate that Pho1 and Dpe1 might form a metabolon in which maltotetraose does not undergo free diffusion, resulting in MOS metabolism with high efficiency.

## Materials and methods

### Cell lysates of sweet potato storage roots

Sweet potato storage roots [*Ipomoea batatas* (L.) Lam. cv Tainong 57] obtained from Sanzhi organic farm in New Taipei City, Taiwan, were ground into powder with liquid nitrogen in a mortar. The ground powder (20 g) was extracted with 10 mL of extraction buffer containing 100 mM Tris-HCl (pH 7.4), 5 mM EDTA, 10 mM β-mercaptoethanol, 20 mM NaF, 1 mM Na_3_VO_4_, 1% (w/v) polyvinylpolypyrrolidone, 1 mM phenylmethylsulfonyl fluoride, complete protease inhibitor cocktail (Roche Diagnostics Ltd.), and 150 mM NaCl followed by centrifugation at 48,000*g* for 30 min at 4°C to obtain the supernatant.

## Antibody

The mouse conventional antisera against Pho1 (αPho1) were prepared as described previously [13]. The mouse monoclonal antibody H7c against Pho1 was prepared as described previously [25]. For generation of the mouse mAb Y1F against sweet potato storage root Dpe1, two Balb/c mice were immunized four times in a two-week interval with the partially purified Dpe1 obtained from the SDS-PAGE where the PD complex was analyzed. The procedures for screening the hybridoma were performed as described previously [25]. The animals’ care and use protocol have been reviewed and approved by the Institutional Animal Care and Use Committee (IACUC) of National Taiwan University (IACUC Approval Number: NTU-98-EL-39). The animals were monitored with daily observation for checking the health condition based on the specific criteria of body weight, body condition score, weakness to obtain feed or water, fight wounds, mobility and activity issues, infection, dermatitis, and tumors. During the experimental period, no Balb/c mice were ill or died. The mice were sacrificed with CO_2_ in an appropriate chamber at the end point of the experiment.

### Immunoprecipitation

Anti-Pho1 poly-clonal antibody or Pho1-specific monoclonal antibody H7c was incubated with protein A Sepharose CL-4B beads (GE Healthcare) on a rotary shaker at 4°C overnight, and then used to immunoprecipitate Pho1 and its potential interacting proteins from sweet potato storage root lysates pretreated with 5 U/mL of amyloglucosidase (EC 3.2.1.3; Sigma-Aldrich product no. A7420, from *Aspergillus niger*) and 7.7 U/mL of α-amylase (EC 3.2.1.1; Sigma-Aldrich product no. A6255, from porcine pancreas) at 25°C for 20 min. Monoclonal antibody Y1F against Dpe1 was used to pull-down Dpe1 and its potential interacting proteins. Controls were performed by replacing anti-Pho1 antibodies or anti-Dpe1 antibody with unrelated mAb (mouse mAb against hemagglutinin). The beads were washed several times with phosphate buffer saline (PBS) containing 0.05% (v/v) Tween-20 (PBST). Bound proteins were eluted by SDS-PAGE sample buffer without containing a reducing agent and subsequently separated by 12.5% SDS-PAGE under a reducing condition.

### Gel electrophoresis and immunoblotting

SDS-PAGE and non-denaturing PAGE were performed according to the method described by Laemmli [26]. After electrophoresis, proteins were either stained with Coomassie Brilliant Blue R-250 (CBR) or transferred to the PVDF membranes (Immobilon-P, Millipore) for western blotting. The protocols were described in details in our previous work [25].

### Identification of proteins by Q-TOF-MS

The procedures for identification of proteins by Q-TOF-MS were performed according to a method described previously [13].

### Immunohistochemistry and confocal spectral microscopy analysis

Specimen preparation was performed according to the method described by Brisson et al. (1989) with a slight modification [27]. Fresh sweet potato storage roots were cut into 1 mm^3^ cubes and fixed in 4% (w/v) paraformaldehyde and 1% (v/v) glutaraldehyde in 50 mM sodium cacodylate with 60 mM sucrose (pH 7.2) at 4°C overnight. Samples were washed three times with cacodylate buffer, dehydrated in a series of ethanol solutions (35%, 50%, 70%, and 100% (v/v)) for 2.5 h in total, and then infiltrated with the London Resin (LR) White resin. Specimens were embedded in 100% resin in gelatin capsules and casted at 50°C in the oven for 24 h. Subsequently, specimens were cut into 1 μm-thick slices, collected on poly-L-Lys-coated slides and treated with 1.7% (v/v) H_2_O_2_ in PBS for 3 min at room temperature. Specimens were further blocked with 5% (w/v) BSA and 10% (v/v) preimmune serum in PBS at 4°C for 12 h. The specimens were finally incubated with Y1F antibody solution (1:10 in PBS with 5% (w/v) BSA) and Cy5-conjugated donkey anti-mouse IgG antibody solution (1:200 in PBS with 5% (w/v) BSA) (Jackson Immunoresearch, West Grove, PA). After washing nine times with PBST, the specimens were blocked again with 5% (w/v) BSA and 10% (v/v) preimmune serum in PBS at 4°C, and then incubated with DyLight 549-conjugated H7c (diluted 1:10 in PBS with 5% (w/v) BSA). DyLight 549-conjugated H7c was prepared by using the DyLight 549 antibody labeling kits (Thermo Scientific, Rockford, IL). Finally, after washing nine times with PBST, specimens were mounted with SlowFade Gold antifade reagent (Molecular Probes, Eugene, OR) to suppress photobleaching and preserve the signals. The fluorescent images were detected by confocal laser scanning microscopy (TCS-SP5, Leica) and analyzed by LAS AF software (Leica). The images of the samples from different developmental stages of sweet potato storage roots were collected using identical parameters. Control experiment was performed by using preimmune serum for comparison.

### Size exclusion chromatography

Size exclusion chromatography was performed by using a Superose 6 10/300 GL column and an AKTA FPLC system (Amersham Pharmacia Biotech). The column was pre-equilibrated with the buffer containing 100 mM Tris-HCl, pH 7.4, 10 mM β-mercaptoethanol, 5 mM EDTA, 20 mM NaF, 1 mM Na_3_VO_4_, 1 mM PMSF, and 150 mM NaCl, and run at a flow rate of 0.25 mL/min. Protein extracts of 10-week sweet potato storage roots (2.5 mg protein) were loaded onto the column in a final volume of 0.5 mL, and fractions of 0.5 mL were collected using a Frac-900 fraction collector (Amersham Pharmacia Biotech). The fractions were separated by 7.5% Native-PAGE and analyzed by western blotting with H7c or Y1F, respectively. The column was calibrated using thyroglobulin (669 kDa), apoferritin (443 kDa), β-amylase (200 kDa), albumin (66 kDa), and carbonic anhydrase (29 kDa) as standards (Sigma-Aldrich, MW-GF-1000).

### Purification of the Pho1-Dpe1 complex and free form Pho1

The PD complex obtained from the high molecular weight fractions of the size exclusion chromatography were further separated on a DEAE-Sephacel column with a linear gradient of NaCl (0.15 M to 0.5 M) in 50 mM Tris-HCl (pH 7.4) buffer. The fractions containing the PD complex were collected and concentrated by ultrafiltration (Microcon YM30, Millipore, Bedford, MA, USA). Pho1 purification was performed according to the method described previously [25]. All purification procedures were carried out at 4°C. Protein content was determined by Bradford dye-binding method [28] (Bio-Rad Protein Assay Kit). Bovine serum albumin (BSA) was used as the standard.

### Pho1 activity assay

Pho1 activity assay in the phosphorolysis directions was carried out according to the procedures described previously [29] with a slight modification. Samples were incubated with 0.5 mM of MOSs (G_1_, G_2_, G_3_, G_4_, G_5_, G_6_, or G_7_), 0.5 mg/mL dextrin, or 0.5 mg/mL amylopectin, respectively, in 50 mM sodium acetate buffer (pH 6.0) containing 0.5 mM inorganic phosphate at 37°C for 1 h. The enzyme reactions were terminated by heating at 100°C for 3 min. Samples were cleared by centrifugation, and the reaction product Glc-1-P in the supernatant was assayed by measuring the amount of NADPH produced by the standard phosphoglucomutase-Glc-6-P dehydrogenase reaction as described previously [29].

### Dpe1 activity assay

The Dpe1 activity assay was performed according to the method described previously [22] with a slight modification. Samples were incubated with 0.5 mM of MOSs (G_1_, G_2_, G_3_, G_4_, G_5_, G_6_, or G_7_), 0.5 mg/mL dextrin, or 0.5 mg/mL amylopectin, respectively, in 50 mM sodium acetate buffer (pH 6.0) at 37°C for 1 h. The enzyme reactions were terminated by heating at 100°C for 3 min. Samples were cleared by centrifugation, and the reaction product Glc was assayed by measuring the amount of NADPH produced by the standard hexokinase-Glc-6-P dehydrogenase reaction as described previously [22].

## Results

### Co-immunoprecipitation of Pho1 and Dpe1 in cell lysates

**Fig 1:**
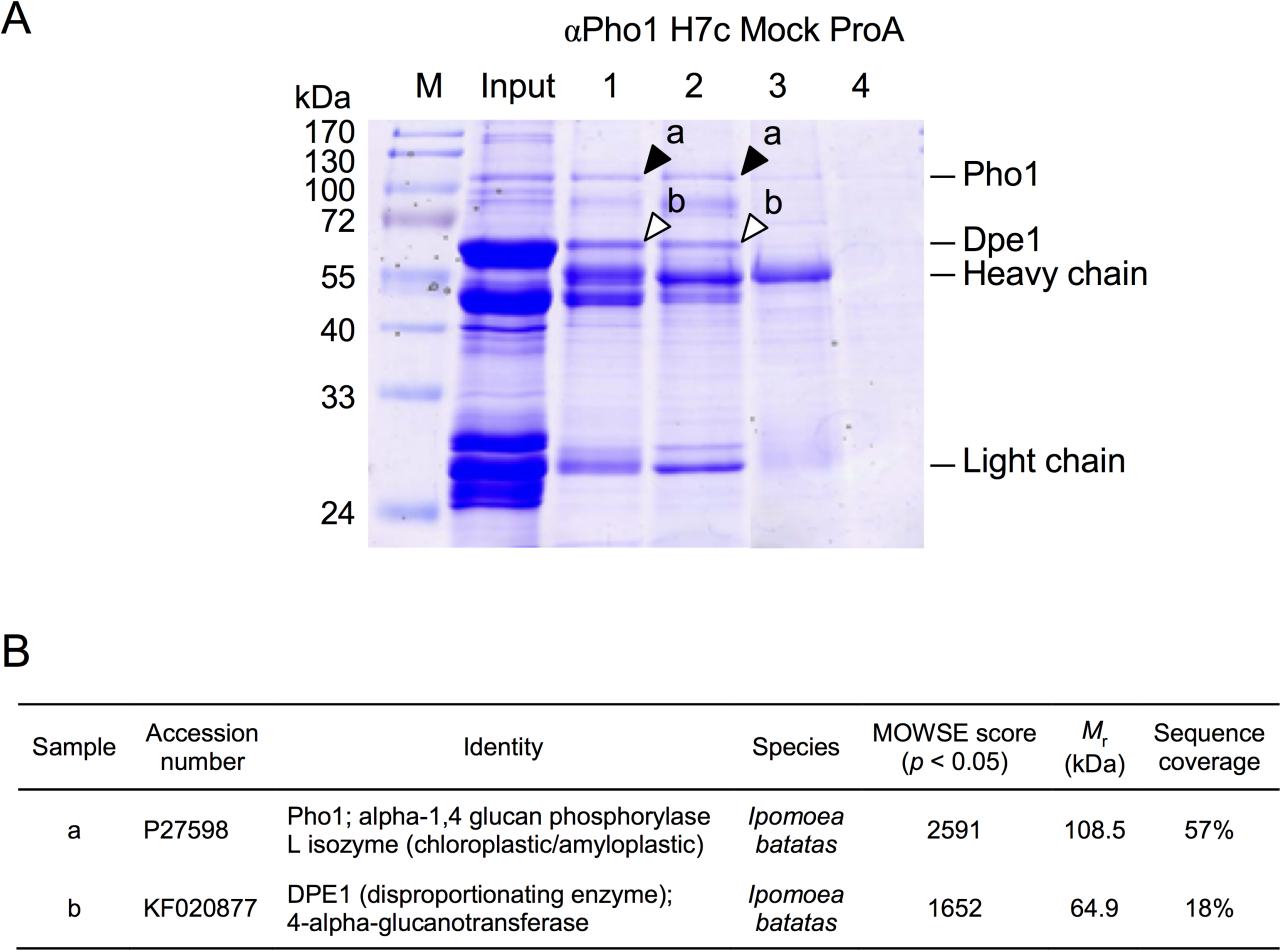
Dpe1 was co-immunoprecipitated with Pho1 by using anti-Pho1 antibody. (A) Whole cell extracts from the 10-week sweet potato storage roots (protein concentration 1 mg/ml) were pre-incubated with glucan-degrading enzymes (5 U/mL of amyloglucosidase and 7.7 U/mL of α-amylase) at 25°C for 20 min, and then incubated with antibodies for Protein-A Sepharose immunoprecipitation: lane 1, conventional antisera against Pho1 (αPho1); lane 2, Pho1 specific mAb (H7c); lane 3, an unrelated mAb (Mock); lane 4, the control experiment using Protein A-Sepharose beads alone without any antibody (ProA). After the immunoprecipitation procedures, the eluted proteins were directly analyzed on 12.5% SDS-PAGE, and stained with Coomassie Brilliant Blue R-250 (CBR). For comparison, 10 μg of the whole cell extracts were also analyzed on the same SDS-PAGE (designated as Input). Arrowheads indicated the positions of Pho1 (closed) and Dpe1 (open). M, pre-stained SDS-PAGE molecular mass standards. (B) The protein samples from bands a and b were sliced out for mass spectrometry analysis. The results demonstrated that band a is Pho1 and band b is Dpe1.

To identify the proteins interacting with Pho1, sweet potato storage root lysates were incubated with anti-Pho1 polyclonal antibody or Pho1-specific monoclonal antibody (mAb) H7c for the immunoprecipitation experiments. Surprisingly, a 65-kDa protein band was simultaneously co-immunoprecipitated with the 110-kDa Pho1 band on the SDS-PAGE (Fig 1A, lanes 1&2). The 65-kDa band was cut out and was subsequently identified as Dpe1 by Q-TOF-MS (Fig 1B).

**Fig 2:**
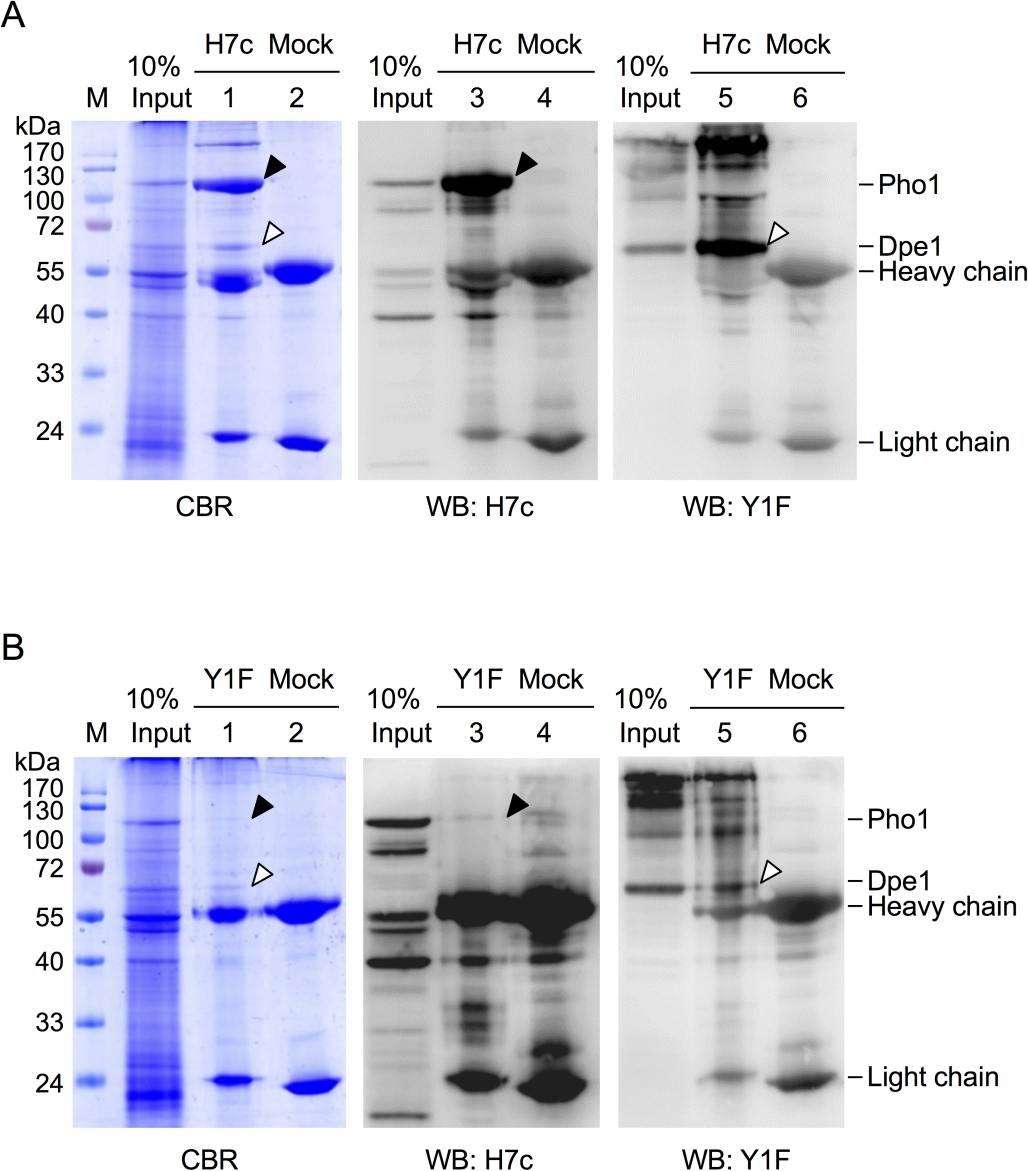
Reverse immunoprecipitation of Pho1 and Dpe1 by specific antibodies. Protein A-Sepharose beads were pre-incubated with Pho1 specific mAb H7c (A) or Dpe1 specific mAb Y1F (B), respectively. Beads were subsequently incubated with the whole cell extracts of sweet potato storage roots which were pre-treated with glucan-degrading enzymes as described in Fig 1. The proteins retained on Protein A-Sepharose were analyzed by 12.5% SDS-PAGE. The gels were stained with CBR, or transferred to PVDF membranes for probing with H7c or Y1F. For comparison, 10 μg of the input cell extracts were also analyzed on the same SDS-PAGE (designated as Input). Controls were performed by replacing H7c or Y1F with an unrelated mAb (Mock). Arrowheads indicated the positions of Pho1 (closed) and Dpe1 (open). M, pre-stained SDS-PAGE molecular mass standards.

To further confirm the interaction between Pho1 and Dpe1, reverse immunoprecipitations were performed by incubating sweet potato storage root lysates with *Ipomoea batatas* Dpe1-specific mAb Y1F, and then followed by SDS-PAGE and western blotting. The results also showed that H7c pulled down both Pho1 (Fig 2A, lane 3) and Dpe1 (lane 5) from cell lysates. On the other hand, Y1F pulled down Dpe1 (Fig 2B, lane 5) and a small amount of Pho1 (Fig 2B, lane 3). These results suggest that Pho1 and Dpe1 might form a protein complex (PD complex) in sweet potato storage roots.

### A high-molecular-weight complex of Pho1 and Dpe1 was isolated on size-exclusion chromatography

**Fig 3:**
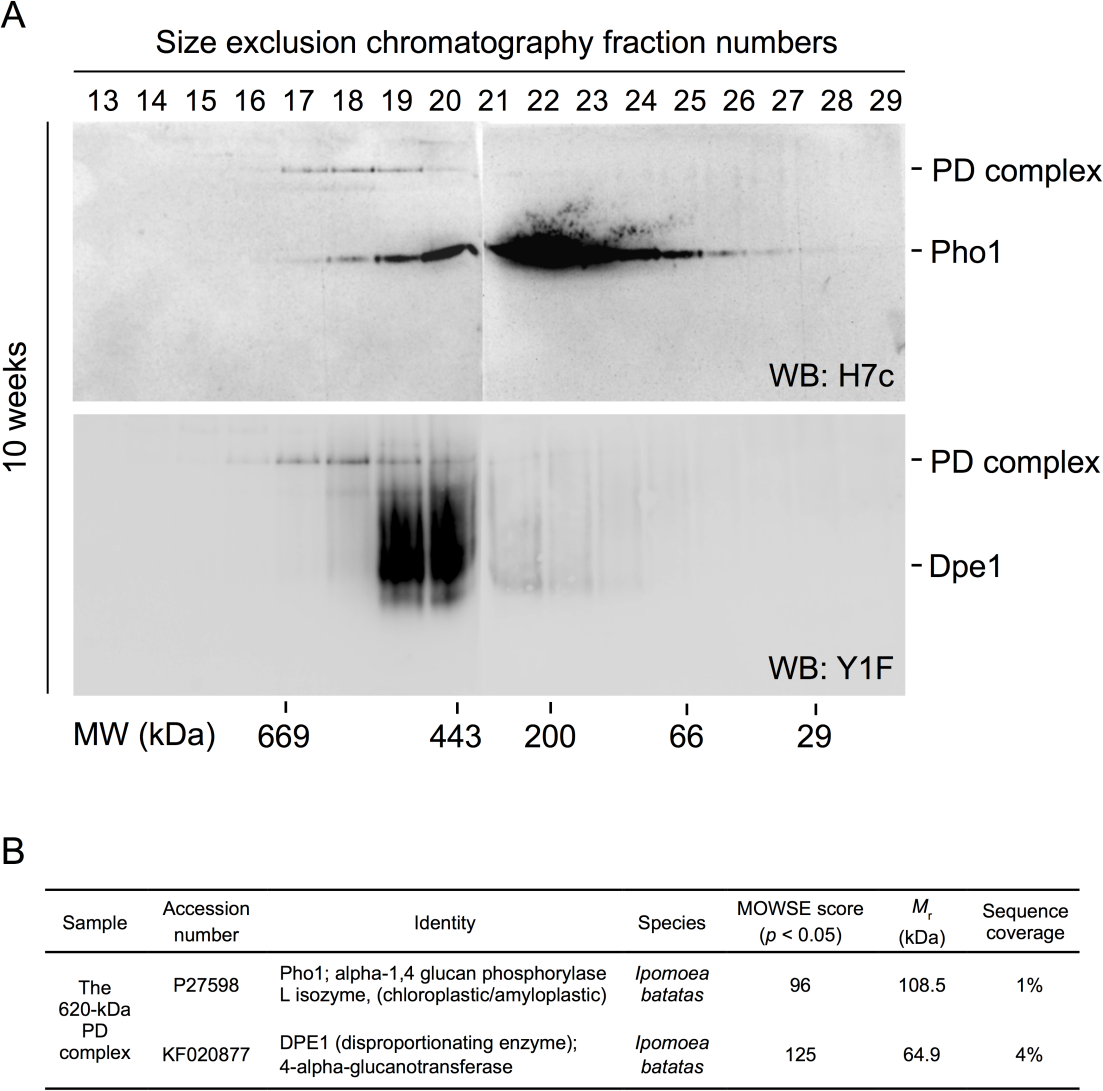
Separation and identification of Pho1, Dpe1, and the PD complex from sweet potato storage root lysates. (A) Cell lysates were prepared from 10-week sweet potato storage roots. The soluble protein extracts (2.5 mg) were separated by Superose 6 10/300 GL size exclusion chromatography. The fractions were analyzed on 7.5% Native-PAGE and then transferred to PVDF membranes for western blotting by using H7c (upper panel) or Y1F (lower panel), respectively. The molecular mass in kilodalton (kDa) was shown as indicated at the bottom of the figure by using the molecular mass standards (thyroglobulin, 669 kDa; apoferritin, 443 kDa; β-amylase, 200 kDa; albumin, 66 kDa; carbonic anhydrase, 29 kDa) analyzed on the same column chromatography condition. (B) The 620-kDa bands, which can be simultaneously probed by H7c and Y1F, were subsequently analyzed by Q-TOF-MS and confirmed as the complex of Pho1 and Dpe1.

In an attempt to purify the PD complex and examine the protein interaction between Pho1 and Dpe1, sweet potato storage root lysates were separated by size exclusion chromatography. Analysis of the eluted fractions by Native-PAGE and immunoblotting with H7c (Fig 3A, upper panel) revealed that Pho1 was distributed in two groups according to their molecular sizes at 620 kDa (fractions 17 to 19) and 220 kDa (fractions 19 to 24). The major bands at 220 kDa were identified as the native homodimeric form of Pho1 [30, 31]. Notably, the parallel immunoblotting experiment with Y1F (Fig 3, lower panel) also revealed high-molecular-weight protein bands at 620 kDa (fractions 17 to 19) in addition to the native 510-kDa Dpe1 bands (fractions 19 to 20). These 620-kDa bands were subsequently analyzed by Q-TOF-MS and confirmed as the complex of Pho1 and Dpe1 (Fig 3B).

### Both Pho1 and Dpe1 were located in sweet potato storage root amyloplasts

**Fig 4:**
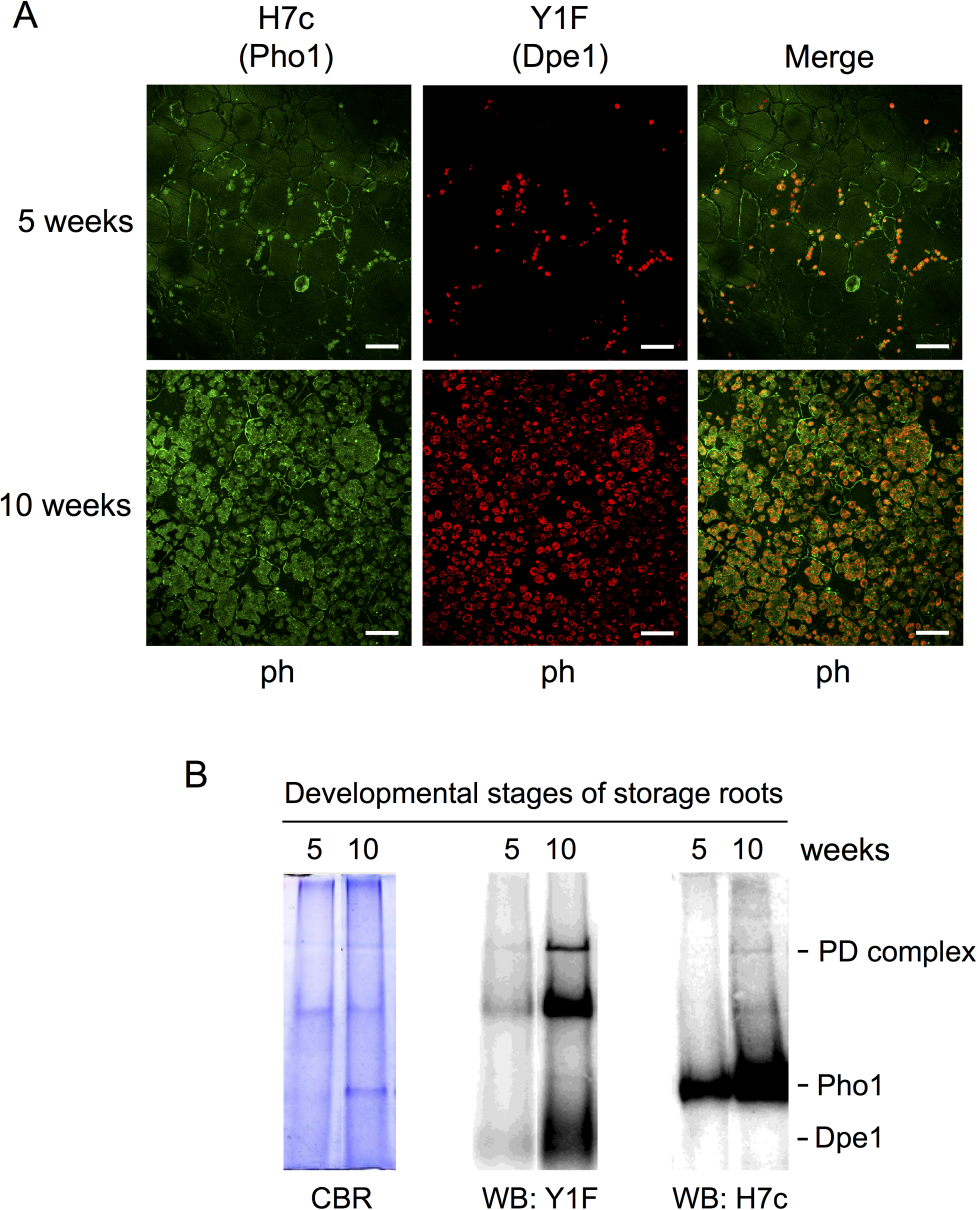
Pho1 and Dpe1 were localized in the amyloplasts of sweet potato storage roots. (A) The histochemical distributions of Pho1 and Dpe1 from two developmental stages of sweet potato storage roots (5 weeks and 10 weeks) were examined by double immunofluorescence labeling and observed by confocal spectral microscopy. Dpe1 was probed with Y1F and then labeled with Cy5-conjugated donkey anti-mouse IgG (shown in red). Pho1 was probed with DyLight 549-conjugated H7c (shown in green). The red-green overlay of the images was shown in yellow revealing the co-localization of Pho1 and Dpe1 in the amyloplasts. The diameters of 5-week and 10-week sweet potato storage roots were 3 and 9 mm, respectively. ph, phloem. White bar, 50 μm. (B) The cell lysates of sweet potato storage roots (5 weeks and 10 weeks) were analyzed on 7.5% Native-PAGE. The gels were stained with CBR, or transferred to PVDF membranes for western blotting by using Y1F or H7c, respectively.

The double immunofluorescence labeling experiments by H7c and Y1F mAbs were used to examine the subcellular localization of Pho1 and Dpe1 at two developmental stages of sweet potato storage roots (5 weeks and 10 weeks). Observation of the antibody-labeled tissue sections by confocal spectral microscope showed that the protein levels of Pho1 and Dpe1 both increased extensively as the sweet potato storage root was fully developed at 10-week stage (Fig 4A). The overlay images further showed that Dpe1 and the majority of Pho1 were co-localized inside the amyloplasts of phloems, and a fraction of Pho1 were found along the cell walls. Previous studies also reported that Pho1 was present in amyloplasts, cytoplasm or cell walls [27, 32, 33]. To further investigate whether the amounts of Pho1 and Dpe1 increase in older tissues, the sweet potato storage roots collected from different developmental stages (5 weeks and 10 weeks) were used to perform the western blotting. The results showed that the amounts of Pho1, Dpe1, and the PD complex increase along with the development of sweet potato storage roots (Fig 4B). In addition to analysis of the amounts of Pho1, Dpe1, and the PD complex at the 10-week developmental stage of the sweet potato storage roots (Fig 3A), the lysates of the sweet potato storage roots at 5-week developmental stage were also analyzed by size exclusion chromatography and the eluted fractions were further analyzed by Native-PAGE and immunoblotting with H7c or Y1F. The results showed that the amounts of Pho1, Dpe1, and the PD complex at the 5-week developmental stage (S1 Fig) were much less than the amounts at the 10-week developmental stage (Fig 3A). These observations suggest that the expression of Pho1 and Dpe1, and the formation of the PD complex are paralleled to the starch accumulation in the developing sweet potato storage roots.

### The Pho1-Dpe1 complex catalyzed Pho1-nonreactable substrates and enhanced Dpe1 activity

**Fig 5:**
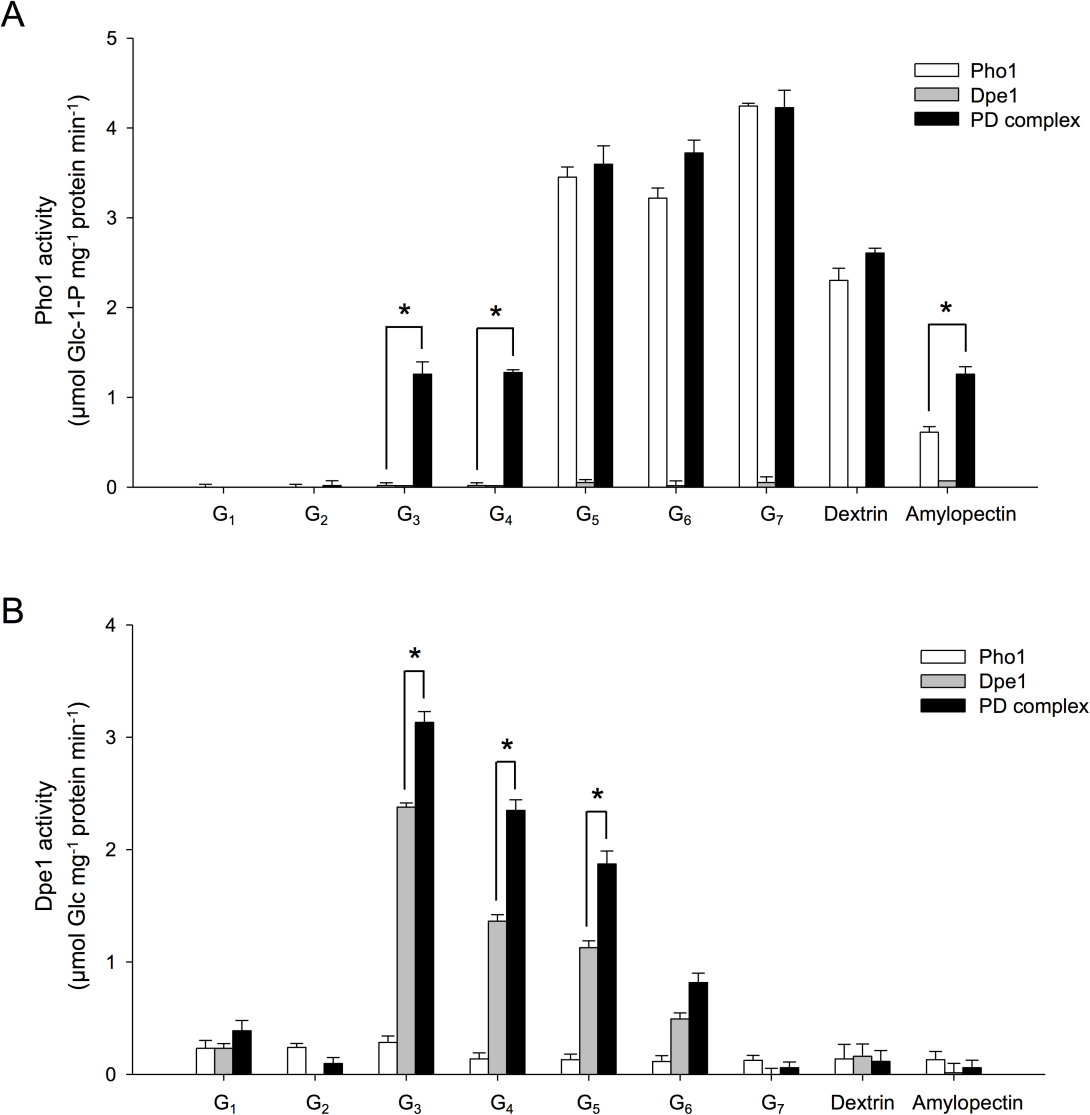
The PD complex displayed higher Dpe1 activities towards maltotetraose than that of free form Dpe1. Pho1 (white bars), Dpe1 (gray bars), or the PD complex (black bars) was incubated with 0.5 mM of MOSs (G_1_, Glc; G_2_, maltose; G_3_, maltotriose; G_4_, maltotetraose; G_5_, maltopentaose; G_6_, maltohexaose; G_7_, maltoheptaose), or 0.5 mg/mL of dextrin or amylopectin, respectively, in the presence (A) or absence (B) of 0.5 mM inorganic phosphate (Pi) in 50 mM acetate buffer (pH 6.0) at 37°C for 1 h. The enzyme activity of Pho1 was denoted as the rate of Glc-1-P production (A). The enzyme activity of Dpe1 was denoted as the rate of Glc production (B). Data represent means ± SD (n = 3). Asterisks indicate statistically significant differences in Pho1 activity (A) or Dpe1 activity (B) of the PD complex (*p* ≤ 0.05, Student's t-test) compared to the free form Pho1 (A) or the free form Dpe1 (B) toward each indicated substrate, respectively.

To determine whether the catalytic behaviors of Pho1 and Dpe1 might be changed while forming a complex, the catalytic reaction rates of Pho1, Dpe1, and the PD complex toward Glc (G_1_), maltose (G_2_), maltotriose (G_3_), maltotetraose (G_4_), maltopentaose (G_5_), maltohexaose (G_6_), maltoheptaose (G_7_), dextrin, or amylopectin were measured, respectively. Fig 5A showed that Pho1 was not reactive toward G_1_, G_2_, G_3_, and G_4_, but displayed high catalytic activity with G_5_, G_6_, G_7_, or dextrin to produce Glc-1-P (white bars) in the presence of inorganic phosphate. The purified Dpe1 did not show any Pho1 activity (grey bars). However, the PD complex displayed moderate Pho1 activity toward G_3_ and G_4_ (black bars), which are not substrates of free form Pho1. Moreover, the catalytic activities of Pho1 and the PD complex toward G_5_, G_6_, G_7_, and dextrin were similar, but the PD complex displayed higher activity toward amylopectin than Pho1 alone (Fig 5A). Together, these observations indicated that the catalytic behavior of Pho1 was changed after forming a complex with Dpe1. On the other hand, Fig 5B showed that the purified Pho1 did not have obvious Dpe1 activity (white bars), and Dpe1 alone was reactive toward G_3_, G_4_, G_5_, and G_6_ with a preference of shorter glucans (grey bars), particularly G_3_ and G_4_. The PD complex also showed the same substrate preference and tendency with Dpe1, but its Dpe1 activity was enhanced markedly (black bars).

To compare the enzyme kinetic parameters of Dpe1 and the PD complex toward G_3_ or G_4_, the initial rates of Glc production at various G_3_ or G_4_ concentrations were measured. The results in Table 1 showed that Dpe1 and the PD complex displayed very similar Michaelis-Menten constant (*K*_m_) toward G_3_ or G_4_, respectively, indicating that Dpe1 did not change substrate preference while forming a complex with Pho1. However, *K*_m_ values for G_3_ are smaller than those for G_4_, suggesting that both Dpe1 and the PD complex prefer to catalyze shorter MOSs. For turnover numbers, Dpe1 and the PD complex showed similar *k*_cat_ for G_3_ (5.58 s^-1^ & 5.90 s^-1^), but have smaller *k*_cat_ for G_4_ (2.81 s^-1^ & 4.51 s^-1^). In terms of G_4_, the PD complex had higher *k*_cat_ (4.51 s^-1^) than Dpe1 (2.81 s^-1^), revealing that Dpe1 displayed approximately 1.5-fold higher catalytic activity upon forming a complex with Pho1. Finally, the *k*_cat_/*K*_m_ value of the PD complex for G_4_ (5.31 mM^-1^ s^-1^) was higher than that of Dpe1 (3.51 mM^-1^ s^-1^). Taken together, these results indicated that the catalytic behavior of Dpe1 was changed after forming a complex with Pho1.

**Table 1:** Enzyme kinetic parameters of the PD complex and the free form Dpe1 toward maltotriose (G_3_) or maltotetraose (G_4_).

	*K*_m_ (mM)		*k*_cat_ (s^-1^)		*k*_cat_/*K*_m_ (mM^-1^ s^-1^)	
	G_3_	G_4_	G_3_	G_4_	G_3_	G_4_
Dpe1	0.68 ± 0.47	0.80 ± 0.34	5.58 ± 2.58	2.81 ± 0.77	8.21	3.51
PD complex	0.68 ± 0.40	0.85 ± 0.11	5.90 ± 3.48	4.51 ± 0.28*	8.68	5.31*

Values are mean ± SD (n = 3). Asterisks indicate statistically significant differences in the enzyme kinetic parameters of the PD complex (*p* ≤ 0.05, Student's t-test) compared to the free form Dpe1.

## Discussion

This study presents evidences for the protein-protein interaction between Pho1 and Dpe1 in the amyloplasts of the developing sweet potato storage roots. From our results, the reciprocal co-immunoprecipitation and size exclusion chromatography experiments demonstrated the physical interaction between Pho1 and Dpe1. A recent study also demonstrated a similar finding, showing that OsPho1 and OsDpe1 form a protein complex in rice endosperm [34].

Many studies found that Pho1 is localized in the amyloplast stroma of wheat and rice endosperm [5, 35, 36]. Our previous study showed that Pho1 is localized in the phloem area at earlier development stages of sweet potato storage roots [33]. It was also reported that Dpe1 is localized within the amyloplasts of wheat endosperm [37]. The immunohistochemistry analysis in this study revealed the co-localization of Pho1 and Dpe1 in the amyloplasts of phloem, and the amount of the PD complex was markedly increased throughout sweet potato storage root growth (Fig 4), suggesting that these two enzymes might have functional coordination and play essential roles in starch metabolism.

Furthermore, Pho1 was reported to be associated with starch synthesis-related enzymes in wheat [14], maize [15], barley [16], and rice [17]. These findings suggest that Pho1 might interact with various partners at various plant developing stages. Previous studies have also mentioned that phosphorylation of Pho1 is part of a mechanism that regulates the formation of the protein complex between Pho1 and different proteins [14, 15, 29, 38]. However, the phosphorylated Pho1 was not found in the PD complex in our mass spectrometry analysis data (data not shown).

Previous genetic studies have shown that repression of *Dpe1* in potato and the *Dpe1* mutant of *Arabidopsis* displayed an increased activity of Pho1 [19, 20]. On the other hand, mutation of *Pho1* gene in *Arabidopsis* caused a loss in Dpe1 activity [39]. Furthermore, Glc-1-P production catalyzed by Pho1 in the presence of G_3_ and G_4_ was significantly higher in the crude extracts of Dpe1 wild-type than the amount derived from *Dpe1* mutants in *C*. *reinhardtii* [6, 7]. Removal of Glc residue from G_3_ substrates by Dpe1 caused a 10-fold increase in Glc-1-P production by Pho1 [22]. These findings revealed the functional linkage between Pho1 and Dpe1. Similarly, we observed that Pho1 cannot generate Glc-1-P by using G_3_ and G_4_ as its substrates, but Glc-1-P was markedly produced when the PD complex was incubated with G_3_ and G_4_ (Fig 5A), implying that the catalytic intermediates generated by Dpe1 was turned into the substrates of Pho1. Therefore, the detectable Pho1 activity in the PD complex toward G_3_ or G_4_ (Fig 5A) was likely due to the presence of longer maltodextrins (G_5_ or larger) generated by Dpe1.

Previous studies indicated that the major substrate for Dpe1 is G_3_ [6–8, 19]. Takaha et al. found that G_3_, G_4_, and G_5_ were effective substrates of Dpe1 [23]. We also found that G_4_ was a favorite substrate for Dpe1 (Fig 5B). Moreover, the PD complex showed higher catalytic activity toward G_4_ over Dpe1 alone (Table 1), implying that the substrate preference of Dpe1 was changed while forming a complex with Pho1.

It is also noted that the phosphorolytic activity of Pho1 in the PD complex is higher than the activity of free form Pho1 while amylopectin is present as a substrate (Fig 5A). Previous studies suggested that the physiological function of Pho1 is to elongate short-chain MOSs, which can serve as primers for the reactions catalyzed by starch synthases or starch branching enzymes [5, 40, 41]. A recent study also proposed that the physiological function of OsPho1 within OsPho1:OsDpe1 complex is to convert G_5_ to longer MOSs [34]. However, as the concentration of inorganic phosphate (Pi) is 50-fold higher than that of Glc-1-P in potato tuber plastids [42], the glucan phosphorolytic activity of Pho1 would be greater than the synthetic activity under the physiological condition. Previous study also found that Pho1 favored the phosphorolytic reaction over the synthetic reaction when MOSs were present [11]. Therefore, a more likely scenario is that Pho1 performs phosphorolysis reaction and the residual product would be elongated by the action of Dpe1 within the PD complex.

Analysis of the kinetic parameters of Dpe1 toward G_4_ showed that Dpe1 within the PD complex has a 1.5-fold larger *k*_cat_ than does the free form Dpe1 (Table 1). Thus, the increased catalytic activity of Dpe1 within the PD complex toward G_4_ might be the result of physical interaction with Pho1. Although the precise structure of the PD complex was not clear, we can speculate that the occurrence of metabolite channeling composed of Pho1 and Dpe1 can protect G_4_ and longer glucan intermediates from free diffusion, and consequently enhance the catalytic efficiency mediated by the PD complex.

## Conclusions

**Fig 6:**
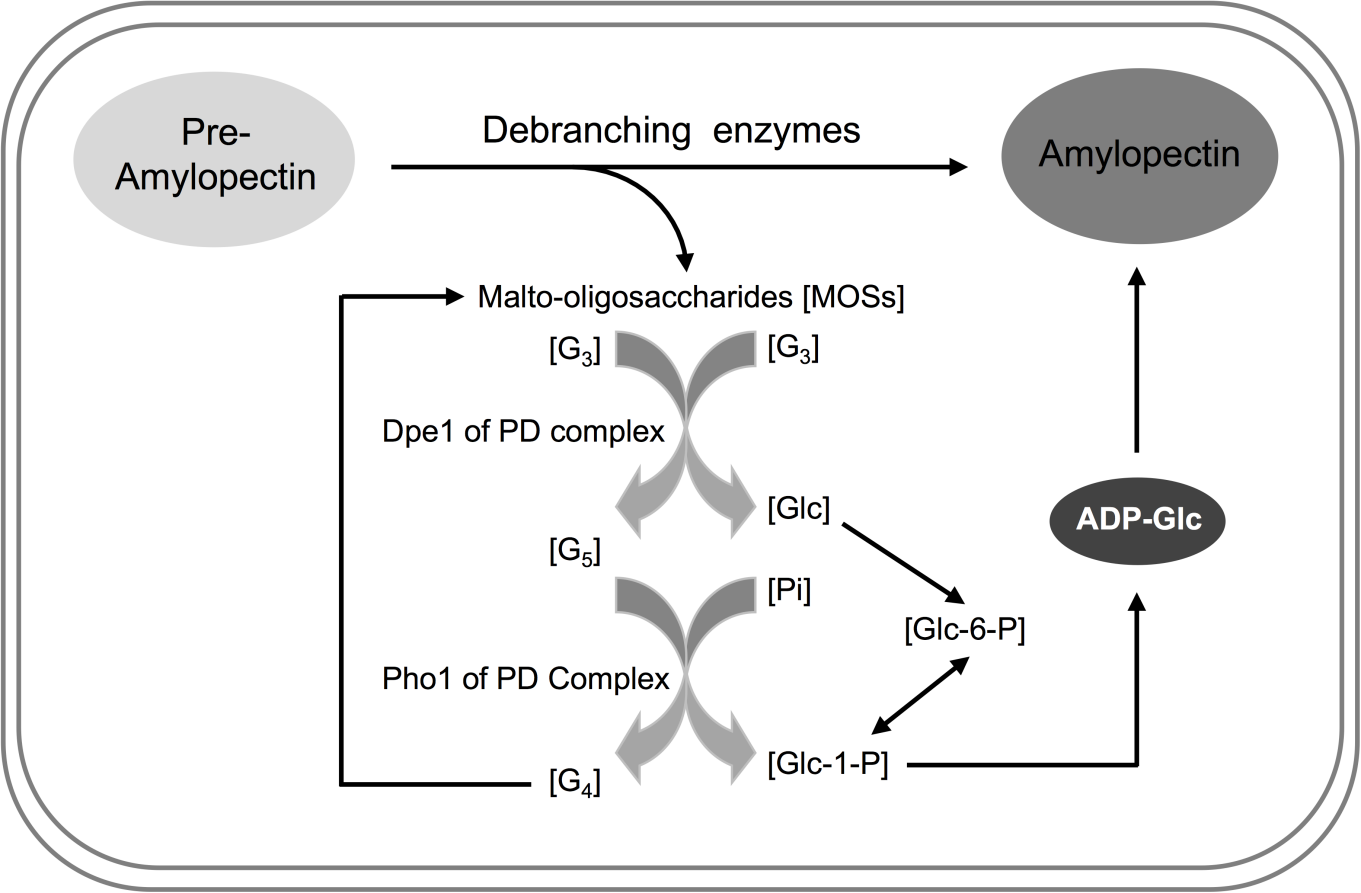
A possible physiological role of the PD complex in amylopectin biosynthesis. In amylopectin biosynthesis process, the wrongly positioned branch glucans are removed by debranching enzymes from pre-amylopectin to release MOSs and therefore promote amylopectin crystallization. While maltotrioses (G_3_) is generated in the process, Dpe1 in the PD complex might utilize G_3_ to generate G_5_ (maltopentaose) and Glc. Subsequently, G_5_ is efficiently catalyzed into G_4_ (maltotetraose) and glucose-1-phosphate (Glc-1-P) in the presence of inorganic phosphate (Pi) by Pho1 in the PD complex. In addition, G_4_ is recycled back to the MOSs pool and is also a suitable substrate for Dpe1 in the PD complex. Glc and Glc-1-P are converted to glucose-6-phosphate (Glc-6-P) and ADP-glucose (ADP-Glc) for amylopectin biosynthesis.

Taken together, we propose that a potential physiological function for the PD complex might serve as a metabolon involved in the recycling of MOSs back into amylopectin biosynthesis. A schematic diagram of the proposed model was illustrated in Fig 6. It is speculated that wrongly positioned branch glucans are removed from pre-amylopectin to promote amylopectin crystallization in amylopectin biosynthesis pathway. MOSs are released by debranching enzymes in this process [1–3] and are suitable substrates for Dpe1. In the PD complex, Dpe1 catalyzes two G_3_ molecules into G_5_ and Glc [43], and then Pho1 catalyzes G_5_ to G_4_ and Glc-l-P. Subsequently, Glc and Glc-l-P are converted to ADP-Glc for amylopectin biosynthesis. Moreover, G_4_ could be recycled back to the MOSs pool and be a favorable substrate of Dpe1. As a result, the PD complex could efficiently maintain the pool of ADP-Glc for starch synthase (Fig 6). When the concentration of ADP-Glc must be increased in amyloplasts, the enhanced activities of the PD complex can provide sufficient Glc-1-P supply by efficiently recycling of MOSs back into amylopectin biosynthesis.

## Supporting information

S1 Fig Separation of Pho1, Dpe1, and the PD-complex derived from the lysates of sweet potato storage roots at the 5-week developmental stage by size exclusion chromatography. The soluble protein extracts (2.5 mg) derived from 5-week sweet potato storage roots were separated by Superose 6 10/300 GL size exclusion chromatography. The fractions were analyzed on 7.5% Native-PAGE and then transferred to PVDF membranes for western blotting by using H7c or Y1F, respectively. The molecular mass in kilodalton (kDa) was shown as indicated at the bottom of the figure by using the molecular mass standards (thyroglobulin, 669 kDa; apoferritin, 443 kDa; β-amylase, 200 kDa; albumin, 66 kDa; carbonic anhydrase, 29 kDa) analyzed on the same column chromatography condition.
(TIFF)
