# Supplementary figures and images for: Plastidial α-glucan phosphorylase 1 complexes with disproportionating enzyme 1 in Ipomoea batatas storage roots for elevating malto-oligosaccharide metabolism
Source: PLoS One. 2017 May 4;12(5):e0177115. doi: 10.1371/journal.pone.0177115 (PMC5417683; doi:10.1371/journal.pone.0177115)

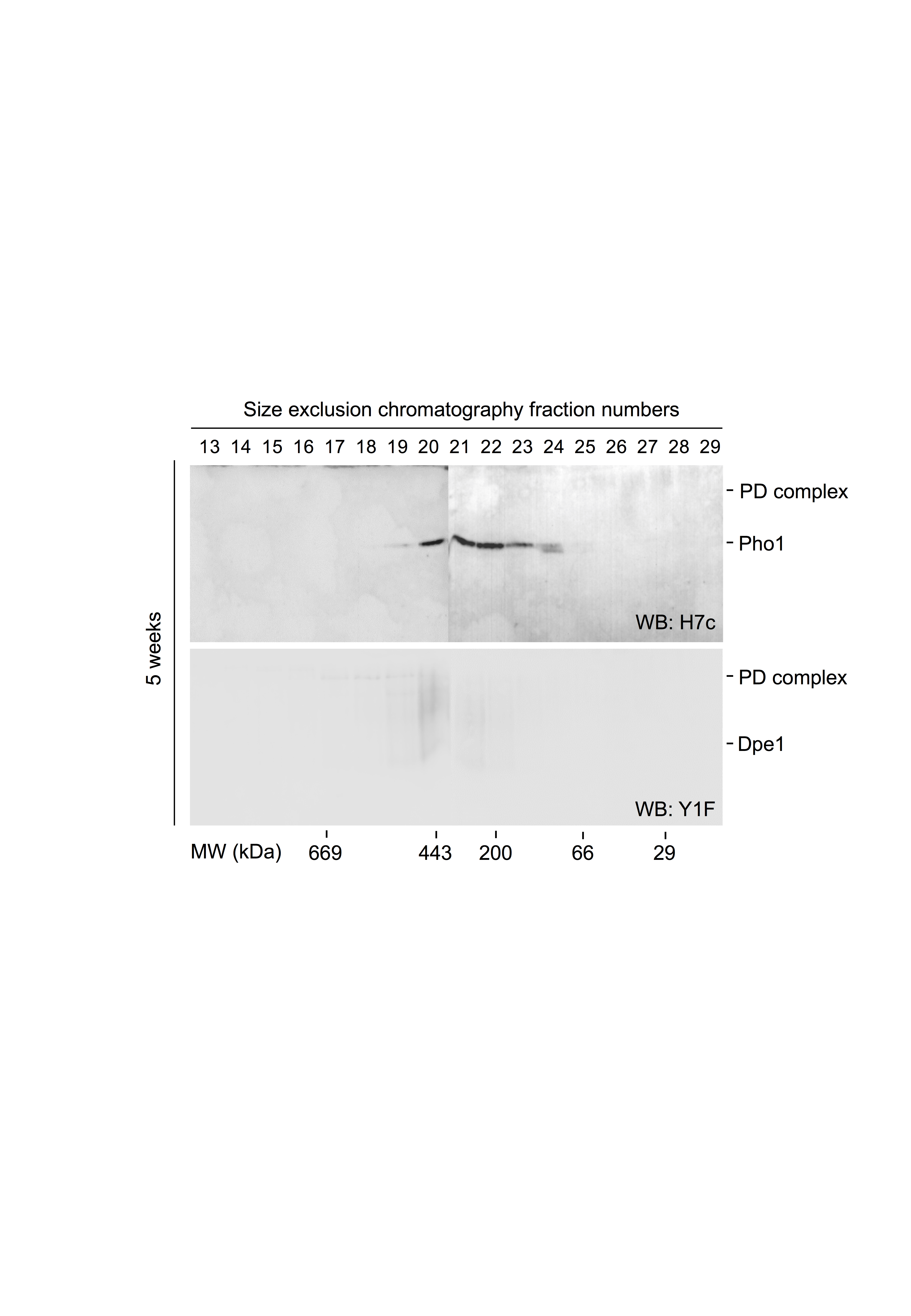

Supplement: S1 Fig — The soluble protein extracts (2.5 mg) derived from 5-week sweet potato storage roots were separated by Superose 6 10/300 GL size exclusion chromatography. The fractions were analyzed on 7.5% Native-PAGE and then transferred to PVDF membranes for western blotting by using H7c or Y1F, respectively. The molecular mass in kilodalton (kDa) was shown as indicated at the bottom of the figure by using the molecular mass standards (thyroglobulin, 669 kDa; apoferritin, 443 kDa; β-amylase, 200 kDa; albumin, 66 kDa; carbonic anhydrase, 29 kDa) analyzed on the same column chromatography condition. (TIFF) [file pone.0177115.s001.TIFF]
